# Not just quantity but also quality of language: Cross-cultural comparisons of maternal mental state talk in New Zealand, Australia, and China

**DOI:** 10.1371/journal.pone.0282480

**Published:** 2023-03-16

**Authors:** Qiuyi Kong, Aisling Mulvihill, Virginia Slaughter, Harry Fraser, Bailey Cavanagh-Welch, Felicia Crysta Elwina, Jie Kang, Ted Ruffman

**Affiliations:** 1 Department of Psychology, The University of Otago, Dunedin, New Zealand; 2 School of Psychology, The University of Queensland, Brisbane, Australia; 3 Department of Mathematics and Statistics, The University of Otago, Dunedin, New Zealand; Tallinn University: Tallinna Ulikool, ESTONIA

## Abstract

Western mothers use more mental state talk with children than do Chinese mothers (e.g., “think”, “like”, “happy”). The present study aimed to examine whether Western mothers not only produced a greater amount of mental state talk, but also used a wider range of mental state terms (i.e., greater lexical variety) compared to Chinese mothers. We compared maternal mental state talk in 271 mother-child dyads from New Zealand, Australia and China, and coded both quantity (i.e., frequency) and quality (i.e., type, variety, valence) of mothers’ mental state talk to their 2.5- to 5-year-olds. Western mothers produced more talk about cognitions and emotions, as well as modulations of assertions, but a similar amount of desire talk, compared to Chinese mothers, with the same patterns found in the variety of talk. Western mothers produced an overall higher amount of mental state talk and a greater variety of mental state terms, but crucially, still produced more MS talk after controlling for the variety. Neither the amount nor the variety of maternal MS talk was correlated with children’s theory of mind. These findings shed light on the diverse ways that mothers construe and describe mental states in different cultures, and highlight the importance of examining different aspects of maternal mental state talk and their impact on children’s theory of mind in future longitudinal studies.

## Introduction

An emerging body of research has suggested that maternal mental state (MS) talk varies across cultures. For instance, Eastern mothers tend to talk more about moral rules and behavioral standards, while Western mothers emphasize independent mental states (desires, beliefs, and opinions) [1–4]. There are two factors that might contribute to this contrast: (1) cultural differences in the salience of mental states [5–7], and (2) linguistic characteristics or functions of languages [8, 9].

While socio-cultural factors have received particular attention, linguistic factors have rarely been investigated. The present study, therefore, aimed to examine these two factors by comparing both the total quantity (i.e., frequency) and quality (i.e., type, variety, and valence) of maternal MS talk across three countries (New Zealand, Australia, and China, with New Zealand and Australia together representing “Western” countries). Unlike previous studies, we directly examined a linguistic factor (i.e., the lexical variety in maternal MS talk) to test whether past reports of more MS talk amongst English-speaking parents (compared to non-Western parents) appeared simply because there were more English MS words appropriate for children than Chinese MS words. We also took into account a potential ingroup bias that might affect the validity of our picture stimuli in which mothers might provide more MS talk for ingroup than outgroup members. Further, we examined the links between different aspects of maternal MS talk and children’s theory of mind (ToM). With these changes, the present study shed novel light on how to explain differences in MS talk from both a linguistic and a cultural perspective, and paved the way for future research on the relation between maternal talk and children’s ToM development.

### Comparisons of maternal MS talk

Comparative studies have consistently shown that differences in MS talk arise across countries, with comparisons particularly plentiful for Westerners versus Chinese. For instance, when children were 19 months, Australian mothers used a similar amount of cognition and emotion language compared to Chinese mothers but more desire language [10], indicating that more talk amongst Australian mothers might be age-appropriate, with desire talk at this age more highly linked to subsequent ToM than other types of MS talk [11]. Likewise, European-American mothers referred more to emotions and thoughts with their 3- and 4-year-olds in English, whereas Chinese immigrant mothers living in America, who mainly or exclusively spoke Chinese, referred more to behavior while telling stories [1, 12, 13]. Similarly, when asked to describe their own children, British mothers made more comments about their 3- to 5-year-olds’ mental states than did their Hong Kong and Japanese counterparts [5, 14].

Prominent differences in particular types of MS talk are also observed across other non-Western cultures. For instance, US mothers used a similar amount of emotion language with their children aged 10 to 15 months, but referred more to cognitions compared to Chilean mothers [15]. In other cases, there is a culture-specific difference in the *type* of MS talk. Thus, Iranian mothers used a higher proportion of desire talk with children aged 36 to 73 months (*M* = 53mos) compared to New Zealand mothers, whereas New Zealand mothers provided more cognitive talk than Iranian mothers [16]. In sum, there is a general trend for English-speaking Western mothers to talk more about mental states, with the Iranian study the lone exception.

In addition to talk type (i.e., cognition, emotion, desire, etc.), several cross-cultural studies have looked at the valence of talk. Fivush and Wang [17] reported that when talking about shared experiences, Chinese mother-child dyads produced more negative words than did American dyads, whereas American dyads engaged more in negotiations of emotional reactions than Chinese dyads (by having extended conversations about their disagreement and the actual emotions children experienced). This study indicates that it is valuable to identify the valence of talk as the emotion words might convey influential cultural information. For instance, in Western cultures, where independence and individual autonomy are at a premium [18], expression of emotions is encouraged to satisfy individual goals and personal needs, whereas Eastern cultures value interdependence and group interest [19], so that negative emotional expressions have more negative connotations and are strictly discouraged as they might be disruptive to social harmony [3].

In short, the comparative studies described above have compared cultures or sub-cultures (Iran, China, Japan, Chinese in the U.S.) that are *collectivist* as opposed to Western *individualism*. Collectivist cultures place an emphasis on a person’s identification within a group, such as a family or country, and the expectations, duties, and roles that fall to being a member of a group [20], and they value stability in relationships over expression of individual rights. Thus, it is these deeper differences in guiding philosophy that likely make mental states either more or less salient and explain why there is less emphasis on the mental states of *individuals* in collectivist cultures.

To summarize, comparative studies have looked at overall MS talk as well as differences in *types* of MS talk (i.e., cognition, desire, emotion, etc.) and the valence of talk (i.e., positive and negative). The general finding is that parents in Western cultures use more MS terms than parents in non-Western cultures. This begs the question of whether Western parents also use a greater variety of MS words, or just use the same words over and over. To date, we are not aware of any studies that have examined the variety of MS words used by parents across cultures, yet this question has theoretical implications that we describe below.

### Cultural versus linguistic interpretations

Most studies have explained cultural differences in parent MS talk by highlighting a distinction in the parenting aims of Eastern and Western parents. Specifically, Western culture, rooted in ancient Greek philosophy, uses language to give explanations to thinking and to convey meanings of thoughts, concepts and beliefs, whereas Eastern culture, inherited from ancient Chinese philosophy, uses language to guide actions and maintain social interactions [21]. In this sense, the differences in MS talk–in both the amount of using mental states and the variety of different MS terms used–might simply reflect the divergent salience of mental states in different cultures linked to collectivist or individualist aims [5–7, 12].

This socio-cultural account as it pertains to MS amount has been widely accepted until the publication of a recent study of Singaporean bilingual mothers directed researchers’ attention to an alternative linguistic interpretation. Drawing on data from Singaporean mothers, Cheng and colleagues [8] found that bilingual mothers produced more cognitive talk (e.g., “think”) in English than Mandarin, and slightly more desire talk (e.g., “want”) in Mandarin than English. The authors attributed these contrasts to the functions and social goals attached to each language. That is, bilingual mothers used English as a working language for business and instruction, and Mandarin as a mother tongue to deliver cultural beliefs and values. However, an alternative explanation might be that English simply includes a wider range of cognitive words suitable for children compared to Mandarin. This lexical interpretation could equally explain the findings of the Singaporean study, as well as the other cross-cultural findings described above, yet so far as we are aware, it has never been investigated.

There are at least three reasons why one might expect different lexical varieties in English and Chinese words that are appropriate for children. First, lexical ambiguity is a characteristic of Chinese mental-state words but not English. For example, Chinese can express wanting, missing, and thinking using a single word, xiang3 (想), with minor or even no change in the contexts [9]. The multi-purpose meanings of words and phrases would result in a narrower range of MS words in Chinese, whereas a similar language phenomenon (i.e., polysemy in MS words) is not found in English. This feature of Chinese could both reduce the variety of MS words parents use, and also, make it either easier (with fewer MS words to learn) or more difficult (because of the ambiguity of individual terms) for Chinese children to learn about mental states.

Second, it is estimated that there are 7,000 unique characters that are used in the Chinese language, which can be combined into 106,230 words, but that with only 2,500 characters one can read 98% of daily written language [22]. In contrast, it is estimated that a school child learning English will know an average of 88,500 distinct words by year 9 (14 to 15 years of age) [23], with roughly double that number present in the Oxford dictionary as words currently in use [24]. Arguably, then, there are more words needed for basic literacy in English (88,500 by age 14) than Chinese (2500 characters), and this difference in overall word number might mean that the Chinese language contains fewer unique words within categories, such as MS words. Further, although the overall mental-state lexicon of Chinese remains unknown, what seems clear is that most Chinese words are not the words one would use with children, or even in daily conversation between adults [25]. For instance,

1. He is frustrated.
2. 他很伤心.

In the *Oxford Dictionary*, the word “frustrated” has several meanings: (a) 灰心的, 气馁的, (b) 失意的, 不成功的, (c) 情感受挫的, etc., but none of them is widely used in daily situations, let alone when talking to young children in Chinese. That is, even if most English words and phrases could find their translation equivalents in Chinese, English provides more choices for daily use. Indeed, there is some evidence that 3- to 6-year-old Chinese children’s MS talk is initially restricted to a few words: the words for “like” and “know”, with “see”, a perceptual term, also being frequent [26]. This suggests that Chinese mothers’ use of MS terms might also be restricted to a few exemplars when talking to their children.

Third, in colloquial language, English includes specified words to differentiate the nuances of feelings and preferences. For instance, English uses a specific word “favorite”, (“preferred”, “beloved”, “special”, etc.) to describe someone or something that is liked most, whereas Chinese adds an adverb “zui4 最 (most)” before a verb “xi3 huan1 喜欢 (like)” to strengthen the preferences. This difference has the consequence of increasing the variety of English desire words relative to Chinese.

To date, so far as we are aware, there has been no study directly examining the lexical variety of MS talk across cultures or languages. The first aim of the present study, therefore, was to address this gap by measuring the variety of parent MS talk, in addition to the aspects that have been previously examined (i.e., total number of MS terms, types of MS terms such as desire or cognitive, and valence). We argue that variety is crucial because (1) it might account for a greater overall MS use in English-speaking mothers, thus providing a linguistic interpretation of the differences as stated above; (2) it might explain the cultural differences in the timing of ToM development, which involves an ability to understand others’ mental states including desires, beliefs, and knowledge, etc. Linguistic research has suggested that both the amount and variety of parental input are important determinants of children’s vocabulary size [27, 28]. Given that ToM is highly correlated with MS vocabularies, it is plausible that a similar situation applies to ToM, with both the variety and overall amount of maternal MS talk playing important roles. Bekar et al. have found a link between the variety of maternal MS talk and children’s socially adaptive behaviours [29]. Nevertheless, their study compared behavior in higher and lower risk children rather than ToM in Western and Chinese children. Thus, our study provided an initial exploration of these questions by (1) identifying the difference in the variety of MS talk between Western and Chinese mothers, (2) examining whether the difference in variety accounted for a greater amount of talk in Western mothers, and (3) linking the differences in frequency and variety to children’s ToM.

### Ingroup bias

Researchers have used a range of methods to measure MS talk including a wordless picture book about a bear [1, 12], mothers’ reminiscing about previous experiences with children [13], mother story completion when given an initial story context [15], maternal descriptions of their own child [5, 14], free play with mothers commenting on the child’s mental states [10], and descriptions of people in photos [16, 30]. This last task presents an opportunity to examine an interesting possibility, that mothers might have a bias to ascribe more mental states to ingroup members than outgroup members [31, 32]. If the photos only presented people from one group but not the other, dehumanization of outgroups [33, 34], such as a reduced attribution of mental life to the outgroup members [31, 32], might result in an artificial disparity in MS talk, with non-Western mothers talking less about mental states simply because the photos depicted ethnically different individuals. Indeed, prior research has found that adults produced more MS talk when describing ingroup compared to outgroup members [35]. Likewise, McLoughlin and Over [36] found that: (1) 5-and 6-year-olds produced more MS talk when describing ingroups than outgroups, and (2) 6-year-olds used a greater variety of MS words when describing their own group. Thus, the present study used photos of both ingroup children or outgroup children in identical contexts. Specifically, we digitally replaced the heads of children depicted in the stimuli to be either Asian Chinese or Western European, thereby maintaining an identical context in the photos for the MS talk about ingroup or outgroup children.

### The present study

The primary aim of the present study was to compare maternal MS talk with regard to quantity (i.e., frequency) and quality (i.e., type, variety, and valence) in Chinese versus Western mothers. Regarding quality, we coded maternal talk for its MS type (e.g., cognition, emotion, desire, and modulations of assertions), variety (i.e., the number of different MS words mothers used), and valence (e.g., positive, negative, and neutral). Based on previous studies, we expected (a) Western mothers to use more MS talk than Chinese mothers, (b) Western mothers to use a greater variety of MS words given our argument that there might be more MS terms appropriate for children in English than Chinese, and (c) Chinese mothers to use more negative MS words [17].

Also, we compared maternal descriptions of ingroup and outgroup children in identical contexts to examine whether mothers used more MS terms with ingroup than outgroup members. We used 15 pictures in two versions, with one showing Chinese children in various contexts expressing emotions, desires, or cognitive states, and another showing Western children. The two booklets were identical except that the faces of Western children were carefully photoshopped onto Chinese children’s bodies. This meant that the contexts of the two sets of pictures were identical, and all that differed was whether Chinese or Western children were shown. This allowed us to (a) compare within each culture talk about ingroup versus outgroup members, with an interest in whether there was more MS talk for ingroup members, and (b) collapse across the stimuli to examine overall MS talk in Chinese versus Western mothers if there was no cultural bias. Further, we examined how the variety of MS talk related to overall MS use. The question of interest was whether past reports of more MS talk amongst English-speaking mothers (as opposed to non-Western mothers) appeared simply because there were more English MS words appropriate for children (i.e., greater variety of MS terms) in English. To answer this question, we measured the average frequency of talk for each unique MS term (i.e., the frequency of MS talk divided by the variety of MS talk). If Western mothers provided more talk on average for each MS term, they produced more MS talk than Chinese mothers even after controlling for the variety.

Last, not only were we interested in examining differences between Chinese and Western mothers, but we were interested in correlations with children’s ToM. Maternal MS talk is a well-studied contributor to children’s ToM [11, 37, 38]. However, most studies only examined the overall amount of MS use, with little attention being paid to the variety. We argue that examining the link between the variety of MS talk and ToM is necessary because a smaller variety in Chinese might account for the difference in the timing of ToM development (e.g., the generally slower onset of ToM in Hong Kong Chinese children who speak Cantonese Chinese relative to English-speaking children [39]) and the difference regarding the order in which children pass different ToM tasks (e.g., Chinese children understanding knowledge-ignorance earlier than diverse beliefs, whereas English-speaking children showing the reverse order [40, 41]).

To summarize, there were three aims in the present study: (1) to compare Western and Chinese mothers’ MS talk in terms of amount, type, variety, and valence when describing pictures of ingroup and outgroup children; (2) to examine whether a higher amount of MS talk in Western mothers was due to more available MS terms (especially those appropriate for daily use with children) in English; and (3) to examine the relations between different aspects of MS talk and children’s ToM in two distinct cultures.

## Method

### Participants

The participants were 79 New Zealand mother–child dyads, 62 Australian dyads, and 139 Chinese dyads. The New Zealanders and Australians together represented “Westerners” (*n* = 141). The data from nine dyads were excluded due to incompletion (7) and unidentifiable audio records (2). The final dataset included 75 New Zealand mother–child dyads (38 girls; *M*_age_ = 3.60 years, range = 2.17–5.33 years; *SD* = .82), 60 Australian dyads (35 girls; *M*_age_ = 3.55 years, range = 2.50–4.92 years; *SD* = .85), and 136 Chinese dyads (62 girls; *M*_age_ = 3.43 years, range = 2.58–5.50 years; *SD* = .55). However, there were 20 Chinese dyads for whom we had incomplete information (mainly maternal education, *n* = 16), reducing the number for some analyses to 116 (*M*_age_ = 3.41).

This sample size was determined using G*Power [42], which indicated that the required sample size was 84 to detect a significant effect using Analysis of Variance (repeated measures, effect size *f* = 0.2, alpha = .05, power = .95, number of groups = 4, number of measurements = 4). Therefore, our sample of 271 should have been sufficient to detect cultural differences.

Participants were recruited through various means, including a database of parents who showed interest in psychological experiments upon the birth of their child, mothers’ groups on social media, and by word of mouth. Data collection took place in cities in New Zealand and Australia in 2020–2021, and China in 2018–2019. Most children came from middle-class neighborhoods, with the majority of mothers having obtained a university bachelor’s degree or beyond (New Zealanders: 89.9%; Australians: 91.7%; Chinese: 91.1%). None of the children had been diagnosed with any language or psychological disorders and all of them were randomly assigned to describe either European children in the pictures or Chinese children, with the two groups matched for number of mothers, children’s age, and children’s gender.

### Materials

#### Child language

We administered the fourth edition of the Peabody Picture Vocabulary Test (PPVT-4) to assess children’s language ability [43] since child language is a correlate of ToM [44] and maternal MS talk [5, 11]. We used the same test items but translated them into Chinese according to the Peabody Picture Vocabulary Test—Chinese Edition (PPVT-C) [45] when assessing children’s receptive vocabulary in China. The maximum raw score possible was 228.

#### Maternal education

We coded maternal education as a measure of socioeconomic status (SES), with 1 = did not complete high school; 2 = completed high school, 3 = diploma/certificate/vocational qualification, 4 = university bachelor’s degree, 5 = postgraduate master’s degree, 6 = postgraduate doctoral degree.

#### ToM tasks

We used Wellman and Liu’s battery [46] to measure children’s ToM abilities (including Diverse Desires, Diverse Beliefs, Knowledge Access, False Belief Location, and False Belief Contents), supplemented with a level-1 perspective taking task [47]. There were two versions of the level-1 task, one in which children saw a piece of paper with a photo of a dog on one side and a cat on the other, and the other in which children saw a photo of a reclining toddler that could be separated with a piece of card such that the child could see either the upper body or the lower body. In each case, the child initially saw the two identities, then while they viewed one identity (e.g., the cat) were asked what a doll could see (e.g., the cat or the dog). The level-1 perspective taking task was added to the Wellman and Liu’s battery to ensure sensitivity for the younger age range of our sample (2.5 to 5 years). By doing this, we included sensitive tasks with increasing difficulties to better detect subtle differences in children’s understanding. Wellman and Liu’s tasks were combined with our own pictures, story books, dolls, or toys to facilitate children’s responses. For instance, in the Diverse Desires task, the experimenter read a tailor-made storybook for the children with a pop-up flap helping them to engage with the story. Similarly, in the False Belief Contents task, we used pictures of a backpack and closet to show objects and locations.

#### Picture describing task

The Picture Describing task was modified from the task developed by Ruffman et al. [48]. The mother was given a booklet that contained 15 pictures in three contexts: 5 positive contexts (in which an agent showed his/her happiness), 5 negative contexts (in which an agent was upset in a certain context), and 5 reaching contexts (in which an agent tried to reach one object with at least one arm stretching out to show his/her desires). The two booklets (one depicting Western children and the other depicting Chinese children) were identical except that the faces of Western children were carefully photoshopped onto the Chinese children’s bodies to create a realistic photograph and control for the context of the pictures.

All the pictures depicted scenarios in which agents expressed emotions, desires, or cognitive states. Mothers were asked to describe the pictures to the child at their own pace. Their talk was audio-recorded, transcribed and later coded offline by four coders (two for English and two for Chinese). The whole task took around 10–20 min (depending on the length of mothers’ descriptions). S1 Table lists examples of English and Chinese MS words.

The coding scheme for the Picture-Describing task was mainly borrowed from Bartsch and Wellman [49], Ruffman et al. [38] and Taumoepeau and Ruffman [11]. We excluded the utterances that were used to attract children’s attention (e.g., “Look”) and that exactly repeated others’ words, because these utterances did not reflect on the mental states or behavior of the characters in the pictures, or because they simply repeated information.

Following Ruffman et al.’s coding scheme [50], we categorized mothers’ MS talk into four types: cognition (e.g., “think”, “know”), desire (e.g., “like”, “want”), emotion (e.g., “happy”, “sad”), and modulations of assertions (e.g., “might”, “perhaps”). The context was used to decide whether an MS utterance genuinely referred to mental states. Thus, uses of “like” to indicate similarity were not coded as MS terms, and uses of “know” as attention grabbers (e.g., “Know what?”) were not coded as MS terms. In Chinese, “*xiang3* (想)” could refer to thinking, wanting, or missing so its coding was dependent on the context.

To examine the variety of talk, we calculated the number of different MS words mothers used in English and Chinese. For instance, mothers who used “want”, “desire”, and “like” would be coded as giving three different desire terms (variety = 3), whereas mothers who only used “want” repeatedly in different contexts would be coded as 1. Also, we coded the valence of emotion talk based on the positive and negative terms mothers used. Only emotion talk (but not other types of MS talk) could be easily categorized into positive (e.g., “happy”, “pleased”, “excited”, “content”), negative (e.g., “sad”, “angry”, “mad”, “anxious”, “disgusted”) and neutral (e.g., “surprised”).

We first transcribed all the utterances that included MS language and marked non-MS utterances for the coders’ reference. We then had one coder code all the records and the second coder code 25%. Both coders worked from the transcripts but also used the audio records to guarantee accuracy. To measure inter-rater reliability, we calculated Cohen’s Kappa (*κ*) with 0 indicating no agreement and 1 indicating perfect agreement. Reliabilities for Chinese mothers were as follows: emotion: *κ* = .83; desire: *κ* = .87; cognition: *κ* = .76; modulations of assertions: *κ* = .81; positive emotion: *κ* = .91; negative emotion: *κ* = .91; neutral emotion: *κ* = 1.00. Reliabilities for Western mothers were as follows: emotion: *κ* = .93; desire: *κ* = .93; cognition: *κ* = .73; modulations of assertions: *κ* = .79; positive emotion: *κ* = .80; negative emotion: *κ* = .87; neutral emotion: *κ* = 1.00.

### Procedure

Children were tested in three countries successively, with data collection taking place in China first, then in Australia, and then New Zealand. Chinese participants were tested in a quiet room of an education center, and New Zealand and Australian mothers and children were tested in laboratories at the Psychology Departments. After greeting mothers and children, the experimenter provided mothers with an informed consent form and a questionnaire regarding demographic information. While mothers were filling in the questionnaire, the experimenter warmed up with children. For all participants, the language test (i.e., PPVT-4) appeared first to help children get familiar with the environment and the process. Children then received six ToM tasks in a pseudorandomized order. In all orders, Diverse Desires and Diverse Beliefs were grouped together and Knowledge Access, False Belief Location, and False Belief Contents were grouped together, with Perspective Taking as the third group. The order of tasks was first randomized in each group, and the order of the groups was randomized. Lastly, the experimenter gave the Picture-Describing task. In this task, mothers were asked to sit on a chair with the child on her lap and were left in the room alone. While the experimenter waited outside, mothers described the pictures to their children as if they were at home reading a story, with the procedure audio-recorded. The whole experiment took around 45 minutes.

This study received ethical approval from the University of Queensland and University of Otago Human Ethics Committee (2019001955 and 21/110). Informed consent was obtained from all participants to make sure they understand the purpose, eligibility criteria, and procedure of the study prior to beginning.

### Inclusivity in global research

Additional information regarding the ethical, cultural, and scientific considerations specific to inclusivity in global research is included in the Supporting Information (S1 Checklist).

## Results

### Analytic plan

We first compared Western and Chinese MS talk in terms of amount, type, variety, and valence when describing pictures of Western and Chinese children. We then examined whether Western mothers’ greater use of MS talk was due to more available MS words in English by comparing the group differences in the average frequency of talk for each unique MS term (i.e., the frequency of MS talk divided by the variety of MS talk). Lastly, we examined the correlations between different aspects of maternal MS talk (i.e., amount and variety) and children’s ToM in two distinct cultural groups.

### Descriptive statistics

Table 1 presents descriptive statistics for the key variables in New Zealand, Australia, and China. We first computed a between-subjects analysis of variance (ANOVA) with country as the independent variable and child age as the dependent variable to check if the ages were similar in the three countries. Child age did not differ across countries, *F*(2, 268) = 1.55, *p* = .21, *η*_*p*_^*2*^ = .01. Then, we used ANOVA to examine maternal education levels and found a significant difference, *F*(2, 252) = 5.73, *p* = .004, *η*_*p*_^*2*^ = .04. Subsequent independent groups *t*-tests revealed that there was no difference between New Zealand and Australian mothers, *t*(133) = 0.52, *p* = .61. Therefore, we collapsed the New Zealand and Australian samples to represent Westerners, as opposed to Chinese, in the following analyses, and later controlled for maternal education to offset the difference that did exist.

**Table 1:** Descriptive statistics for the key variables in three countries.

	Western			China (*n* = 136)
	New Zealand (*n* = 75)	Australia (*n* = 60)	Total (*n* = 135)	
	*M* (*SD*)	*M* (*SD*)	*M* (*SD*)	*M* (*SD*)
Age (years)	3.60 (0.82)	3.55 (0.85)	3.58 (0.83)	3.43 (0.55)
Maternal Education	3.85 (1.11)	3.95 (1.03)	3.90^c^ (1.07)	3.50^c^ (0.78)
Child Language	49.40 (24.19)	76.23 (27.69)	61.33^c^ (28.98)	38.74^c^ (16.08)
Child ToM	2.29 (1.44)	3.13 (1.80)	2.67 (1.66)	2.45 (1.31)

*Note*. Child Language scores are the raw scores from the PPVT with a maximum possible score of 228. The total score for child ToM was 6. For each measure, we compared Western and Chinese data using independent samples *t-*tests, with the Western sample always having higher scores. Superscripts indicate a significant difference and the level of significance: ^c^*p* < .001.

### Amount of maternal MS talk

Table 2 provides the descriptive statistics for each type of mothers’ MS talk in the Picture Description task. We conducted a 2 (Culture: Western, Chinese) × 2 (Picture Type: Western, Chinese) × 4 (Talk Type: cognition, desire, emotion, modulations of assertions) mixed analysis of covariance (ANCOVA), with child age, gender, language ability and mothers’ education levels as covariates, and frequency of mothers’ MS talk as the dependent variable. For some comparisons, Mauchly’s Test of Sphericity indicated that the assumption of sphericity had been violated so we applied the Greenhouse-Geisser correction to our results where appropriate. There were significant effects for Culture, *F*(1, 245) = 97.00, *p* < .001, *η*_*p*_^*2*^ = .284 (with Western mothers producing more MS talk), Talk Type, *F*(2.44, 597.55) = 5.44, *p* = .002, *η*_*p*_^*2*^ = .022, and a Culture × Talk Type interaction, *F*(2.44, 597.55) = 52.43, *p* < .001, *η*_*p*_^*2*^ = .176. All other effects were not significant, all *F*s < 1.91, all *p*s > .167.

**Table 2:** Descriptive statistics for each type of MS language in Westerners versus Chinese.

	Western Mothers			Chinese Mothers		
	Total	Western Pictures	Chinese Pictures	Total	Western Pictures	Chinese Pictures
	*M* (*SD*)	*M* (*SD*)	*M* (*SD*)	*M* (*SD*)	*M* (*SD*)	*M* (*SD*)
Frequency of MS Talk						
Cognition	17.01 (11.66)	16.72 (11.52)	17.32 (11.89)	2.71 (4.40)	1.26 (1.49)	4.07 (5.63)
Desire	5.80 (4.59)	5.94 (4.60)	5.65 (4.61)	5.14 (4.42)	4.60 (3.80)	5.66 (4.90)
Emotion	11.10 (6.64)	10.57 (6.01)	11.67 (7.21)	4.60 (4.07)	3.56 (3.40)	5.57 (4.41)
Modulations of Assertions	6.25 (5.01)	6.38 (4.89)	6.12 (5.15)	0.87 (1.64)	0.65 (1.32)	1.08 (1.87)
Total Frequency	40.17 (21.85)	39.61 (20.99)	40.76 (22.87)	13.02 (9.78)	10.27 (6.39)	16.26 (12.28)
Variety of MS Talk^1^						
Cognition	3.23 (1.54)	3.38 (1.72)	3.08 (1.32)	1.35 (1.39)	1.07 (1.01)	1.68 (1.68)
Desire	2.21 (1.15)	2.29 (1.23)	2.12 (1.07)	1.82 (1.01)	1.72 (1.01)	1.94 (1.01)
Emotion	4.14 (1.84)	3.96 (1.82)	4.33 (1.86)	1.80 (1.46)	1.58 (1.40)	2.05 (1.50)
Modulations of Assertions	2.07 (1.20)	2.13 (1.15)	2.00 (1.25)	0.51 (0.74)	0.36 (0.61)	0.69 (0.84)
Total Variety	11.367 (4.00)	11.79 (4.17)	11.53 (3.83)	5.49 (3.01)	4.74 (2.44)	5.35 (3.37)
Frequency of MS Talk Per Term^2^	3.35 (1.17)			2.54 (1.78)		
Valence of Emotion Talk^3^						
Positive	4.82 (3.57)	4.12 (3.20)	5.56 (3.80)	2.10 (2.07)	1.71 (2.03)	2.56 (2.04)
Negative	4.97 (3.66)	4.94 (3.36)	5.00 (3.98)	1.30 (1.84)	1.07 (1.67)	1.57 (2.00)
Neutral	1.22 (1.92)	1.35 (1.92)	1.09 (1.93)	0.93 (1.29)	0.72 (1.13)	1.18 (1.43)

*Note*.

^1^Variety of talk was calculated as the number of unique MS terms.

^2^Frequency of MS talk per term was calculated as the frequency of MS talk for each unique MS term (i.e., frequency of MS talk ÷ variety of MS talk).

^3^Valence of emotion talk was calculated as the frequency of positive, negative, and neutral emotion utterances.

To further explore the Culture × Talk Type interaction, we examined the effects of Culture on each type of mothers’ talk using a series of ANCOVAs. We covaried out the effects of child age, gender, and language ability (to control for the possibility that aspects of the child resulted in cultural differences), and maternal education (to control for the possibility that mother talk was a function of education). Western mothers used more cognitive talk, *F*(1, 247) = 97.24, *p* < .001, *η*_*p*_^*2*^ = .282, emotion talk, *F*(1, 247) = 62.83, *p* < .001, *η*_*p*_^*2*^ = .203, and modulations of assertions, *F*(1, 247) = 82.46, *p* < .001, *η*_*p*_^*2*^ = .250, than Chinese mothers (see Table 2). However, there was no difference in Western and Chinese mothers’ use of desire talk, *F*(1, 247) = 0.40, *p* = .528.

We then re-ran the 2×2×4 ANCOVA with the proportion of MS talk as the dependent variable given the potential impact of verbosity (i.e., total talk) on MS talk. Chinese mothers might produce less total talk than Western mothers, yet their MS talk could still be equal as a proportion of total talk. Therefore, it was necessary to measure both the frequency and the proportion of MS talk (e.g., the frequency of cognitive talk divided by the frequency of total talk) to rule out the possibility that the cultural differences were driven by the verbosity. The results yielded a very similar pattern, with Western mothers producing higher proportions of cognitive, emotion, and modulation of assertion talk (but not desire talk) and showing no difference in their descriptions of Western and Chinese pictures compared to Chinese mothers. Therefore, we continued to use frequency to represent the amount of talk in the following analyses.

### Variety of maternal MS talk

Next, we conducted a 2 (Culture) × 2 (Picture Type) × 4 (Talk Type) mixed ANCOVA with child age, gender, language ability, and mothers’ education as covariates, and *variety* of mothers’ MS talk as the dependent variable. There were significant effects for Culture, *F*(1, 244) = 114.94, *p* < .001, *η*_*p*_^*2*^ = .320 (with a greater variety of MS terms for Western mothers), Talk Type, *F*(2.53, 616.62) = 4.49, *p* = .007, *η*_*p*_^*2*^ = .018, and Culture × Talk Type, *F*(2.53, 616.62) = 21.77, *p* < .001, *η*_*p*_^*2*^ = .082. All other effects were not significant, all *F*s < 2.55, all *p*s > .111. We then further examined the interaction using a series of ANCOVAs. After controlling for child age, gender, language ability, and mothers’ education, Western mothers used a greater variety of cognitive terms, *F*(1, 246) = 65.24, *p* < .001, *η*_*p*_^*2*^ = .210, emotion terms, *F*(1, 246) = 70.80, *p* < .001, *η*_*p*_^*2*^ = .223, and modulations of assertions, *F*(1, 246) = 94.94, *p* < .001, *η*_*p*_^*2*^ = .278, but Western mothers used a similar variety of desire terms compared to Chinese mothers, *F*(1, 246) = 2.86, *p* = .092.

### Valence of emotion talk

We used a 2 (Culture) × 2 (Picture Type) × 3 (Valence: positive, negative and neutral) mixed ANCOVA, with child age, gender, language ability and maternal education levels as covariates, to examine the valence of mothers’ MS (emotion) talk. There were significant effects for Culture, *F*(1, 244) = 68.33, *p* < .001, *η*_*p*_^*2*^ = .219, Picture Type, *F*(1, 244) = 6.19, *p* = .014, *η*_*p*_^*2*^ = .025, Valence, *F*(2, 488) = 4.60, *p* = .011, *η*_*p*_^*2*^ = .018, Culture × Valence, *F*(2, 488) = 28.82, *p* < .001, *η*_*p*_^*2*^ = .106, and Picture Type × Valence, *F*(2, 488) = 3.97, *p* = .02, *η*_*p*_^*2*^ = .016. Subsequent ANCOVAs revealed that Western mothers used significantly more positive terms, *F*(1, 246) = 32.97, *p* < .001, *η*_*p*_^*2*^ = .118, and more negative terms, *F*(1, 246) = 72.22, *p* < .001, *η*_*p*_^*2*^ = .227, but similar levels of neutral terms, *F*(1, 246) = .59, *p* = .444, compared to Chinese mothers after controlling for child age, gender, language ability and maternal education levels. Across both cultural groups, pictures of Chinese children elicited more positive talk, *F*(1, 246) = 7.93, *p* = .005, but similar levels of negative and neutral talk, *p*s > .613, compared to Western pictures. Although this effect was unexpected, we note that equal proportions of Chinese and Western mothers described the Chinese and Western pictures so that this could not have influenced the very large Culture effects seen in the above analyses.

### Number of MS terms as a function of variety

Given that Western mothers provided more MS talk and also a greater variety of MS terms, our next aim was to examine how the variety related to overall MS use. If there is a greater variety of MS terms appropriate for children in English compared to Chinese, then that might also account for the greater overall frequency of MS talk for Western mothers. Therefore, we examined the frequency of MS talk as a function of the variety of terms each mother used (see Table 2: frequency of MS talk ÷ variety of MS talk). The question of interest was whether Western mothers not only provided more overall MS talk and a greater variety of MS terms, but also, provided more talk on average for each MS term. We used a between-subjects ANCOVA with MS talk per term as the dependent variable, Culture (Chinese, Western) as the factor, and child age, gender, language and maternal education as covariates. Western mothers used more MS talk per term on average than Chinese mothers, *F*(1, 244) = 18.54, *p* < .001, *η*_*p*_^*2*^ = .071. This result indicated that it was not just the relative abundance of MS terms in English (i.e., the variety) that accounted for Western mothers’ greater frequency of MS talk, because they also produced more MS talk per term.

### Relations between different aspects of MS talk and children’s ToM

Since Chinese and Western mothers had the same biases (to describe Chinese children in the pictures more positively than Western children), we collapsed the Western and Chinese pictures in the following analysis. Table 3 lists the correlations between variables in Western and Chinese culture. There were significant correlations among children’s age, language and ToM in both Western and Chinese children. However, neither the total frequency of maternal MS talk nor the variety of talk correlated with children’s ToM in the Western or Chinese groups. In addition, the total frequency of maternal MS talk correlated positively with the variety of talk in both cultures.

**Table 3:** Bivariate correlations between child age, maternal education, child language, mothers’ talk and child ToM abilities in Western (Above diagonal) and Chinese children (Below diagonal).

	1	2	3	4	5	6
1. Child Age	-	-.07	.62^c^	.03	.08	.67^c^
2. Mothers’ Education	-.09	-	.07	.18^a^	.24^b^	-.03
3. Child Language	.49^c^	.11	-	.13	.12	.75^c^
4. Mother MS Talk Total Frequency	.06	.13	.10	-	.78^c^	.08
5. Mother MS Talk Total Variety	.00	.25^b^	.16	.62^c^	-	.08
6. Child ToM	.45^c^	.06	.39^c^	.11	.13	-

*Note*.

^a^*p* < .05,

^b^*p* < .01,

^c^*p* < .001.

All tests are two-tailed.

Since children’s age and language ability significantly correlated with ToM in both Western and Chinese groups (see Table 3), we then used partial correlations to examine the relations between maternal MS talk and children’s ToM after controlling for the effects of child age and language. Still, neither the total frequency of maternal MS talk (Western: *r* = -.001, *p* = .992; Chinese: *r* = .085, *p* = .334) nor the variety of talk (Western: *r* = -.024, *p* = .786; Chinese: *r* = .093, *p* = .295) correlated with children’s ToM after partialling out child age and language in either of the cultural groups.

## Discussion

The present study of 271 mother-child dyads from New Zealand, Australia, and China aimed to examine the contributors to Western parents’ greater use of MS talk compared to non-Western parents. There were two key contributions. For the first time, we examined the lexical variety of MS talk in English-speaking and Chinese-speaking mothers to determine whether Western mothers used more MS terms because there was a greater variety of MS terms appropriate for children in English. Meanwhile, we examined the potential cultural bias in the stimuli by comparing mothers’ descriptions of ingroup and outgroup children. Second, we examined the link between different aspects of MS talk (e.g., frequency and variety) and children’s ToM in two distinct cultures.

There are four main findings: (1) Western mothers provided more talk about cognitions, emotions, and modulations of assertions (but not desires) than Chinese mothers, with the same trends present for the variety of MS talk; (2) Western mothers also provided more MS talk per term, indicating that the sheer variety did not account for the greater amount of MS use in Western mothers; (3) both Western and Chinese mothers produced an equal amount and variety of MS talk when describing ingroup and outgroup pictures; and (4) neither the frequency nor the variety of maternal MS talk correlated with children’s concurrent ToM, even after controlling for child age and language ability. Below, we discuss each of these findings in more detail.

### Amount and Variety of maternal MS talk

In line with previous findings [3, 5, 10–12, 51], we found a higher amount of MS talk in Western mothers relative to Chinese mothers, with Western mothers producing more talk about cognitions, emotions, and modulations of assertions, but a similar amount of desire talk. Interestingly, the same pattern was found in the variety of talk, with Western mothers producing a greater variety of cognitive, emotion, and modulation of assertion talk, but a similar variety of desire talk compared to Chinese mothers.

Most importantly, Western mothers not only provided more overall MS talk and a greater variety of MS talk, but also, more talk on average for each unique MS term, and this latter finding has important theoretical implications. More overall MS talk or a greater variety of MS terms in Western mothers could be caused either by the relative salience of mental states in different cultures (which has led to the creation of more MS words in English than Chinese and more emphasis on mental states when mothers talk), or may be independent of salience and strictly be a function of lexical differences in the number of MS words that are appropriate for children. However, because Western mothers used more MS talk even when controlling for the variety of MS words, linguistic restrictions cannot be responsible for the difference compared to Chinese mothers, suggesting that greater salience accounts for more MS talk in Westerners.

### Ingroup bias

The second aim of the present study was to examine whether mothers had a greater tendency to ascribe mental states to ingroup than outgroup children. We found that mothers produced a similar amount and variety of MS talk for ingroup and outgroup children. Thus, our findings were inconsistent with prior reports of more MS talk for ingroup than outgroup members [34, 35]. One explanation of this difference is that we used pictures of infants and young children toward whom adults tend to show higher empathy and less prejudice [52, 53].

### Maternal MS talk and children’s ToM

Last, we found that neither the amount nor the variety of maternal MS talk was correlated with children’s ToM in either of the two cultural groups. Two meta-analytic studies have examined the relation between parent MS talk and children’s ToM, with one reporting a significant effect size of .21, 95% CI [.14, .27] [54] and another reporting a smaller but significant effect size of .15, 95% CI [.09, .21] [55]. The current effect sizes (ranging from .08 to .13) were comparable to that of Tompkins et al. at the lower end [55], but unlike previous findings, our effects were not significant. One possible explanation for the discrepancies is that our cross-sectional design might have reduced the likelihood of achieving significant correlations. In the second meta-analysis, Tompkins et al. found the strongest relations between MS talk and ToM appeared when there was at least a one-year gap in time [55]. Indeed, when reviewing the cross-sectional studies under greater scrutiny, we obtained rather mixed findings. While some reported significant relations between MS talk and concurrent ToM [56], some did not [57]. Even among those that found significant relations, some reported correlations in one cultural group but not the other [11, 15], some found correlations with fathers’ but not mother’s MS talk [58], and some found relations with boys’ ToM but not girls’ ToM [59]. This pattern makes sense because at any given time, a parent might provide lots of talk to their child either because (a) the child’s language and understanding are good, enabling the parent to pitch their talk at a higher level, or (b) the child’s language and understanding are not good, resulting in the parent trying to scaffold development. Within a timepoint, these two trends would act in opposite directions, resulting in low correlations between parent talk and child ToM. However, when examined longitudinally, in both cases parent language input would correlate with later child understanding. Thus, future research could explore quantity versus variety *longitudinally* to better answer the question as to how maternal MS talk relates to children’s ToM.

### Limitations and future directions

Despite the unique contributions of the present study, there were some limitations. First, as stated above, we adopted a cross-sectional design rather than a longitudinal design, which might account for the weak relation between MS talk and children’s ToM. Linguistic research has shown that parents who simplify language initially will accelerate children’s language development [60]. However, this might only be true initially, with both greater variety and overall amount becoming essential to children’s subsequent learning [26, 27]. We argue that a similar situation might hold true for the relation between MS talk and children’s ToM, with a smaller variety of MS terms facilitating learning initially (with fewer and simpler terms to learn) but the introduction of a larger variety of MS terms becoming necessary later (to provide multiple, related but distinct references to mental states and create appropriate scaffolding within the zone of proximal development). Again, longitudinal studies will be needed to resolve these questions.

Second, while the present study indicates the promise of examining both the quantity and quality (i.e., type, variety, and valence) of MS talk, examining other aspects of MS talk might also help to understand cultural differences. To this end, future research could employ a more fine-grained coding of MS talk to further explore what aspects of MS talk matter cross-culturally, for example by examining the referents of MS talk [15], the appropriateness of descriptions [10] or comparing explanatory, causal, and contrastive MS talk versus the simple mention of mental states [61]. Besides, given that the context of MS talk also affects the results [54, 55], future studies might benefit from using other methodologies to elicit and measure parental MS talk (e.g., naturalistic observations, reminiscing, free play, and self-report). Relevant research could not only help explain the weak relation between MS talk and ToM in the present findings, but also add to the understanding of whether the cross-cultural differences in the amount and variety of MS talk hold in other contexts.

Additionally, other than lexical variety, there might be other linguistic features that deserve attention in future research. For instance, most desire and cognitive terms are verbs (e.g., “think” and “want”) [9]. Despite the fact that Chinese mothers’ MS verbs are reduced relative to Westerners, Chinese-speaking children have an earlier acquisition of non-MS verbs compared to English-speaking children [62–64]. Given the link between parent language input and children’s language acquisition [65], this leads to the following question for future research: Do Chinese mothers use more non-MS verbs than Western mothers, even if they use fewer MS verbs? Complicating verb acquisition, English syntax is more complex than Chinese with regard to MS descriptions. For example, English includes markers for a finite complement (e.g., tense and the complementizer “that”) that are not present in Chinese [9], and this might either complicate or enrich the descriptions of mental states in English.

Further, future research could continue to examine both cultural and linguistic influences by assessing bilingual parents within a specified culture (i.e., controlling for culture) or comparing different cultures in which the same language is spoken (i.e., controlling for language).

In sum, our study did not attempt to disentangle language from culture and the same is true for the vast majority of studies described above. However, we call for a co-consideration and an unconfounding of social-cultural and linguistic factors in future comparative studies. Poortinga et al. have described the process of explaining cross-cultural variance as “peeling the onion called culture” [66]. Drawing on this, a comparison of languages within the same culture, or languages other than Chinese and English, might explain more differences to help peel away one more layer of cross-cultural variances.

## Conclusion

The present study provided two unique and theoretically important insights: (1) Western mothers provided more MS talk and also a greater variety of MS terms compared to Chinese mothers; (2) even when the variety of MS terms is controlled, Western mothers talk more about mental states than Chinese mothers.

## Supporting information

S1 Table Examples of MS talk used by Western and Chinese mothers. *Note*. Some Chinese MS words appeared more than once in the table or in different MS types because they are polysemous (Cheng et al., 2020; Tardif & Wellman, 2000).
(DOCX)

S1 Checklist Inclusivity in global research. (DOCX)
