# Supplementary material for: Not just quantity but also quality of language: Cross-cultural comparisons of maternal mental state talk in New Zealand, Australia, and China
Source: PLoS One. 2023 Mar 16;18(3):e0282480. doi: 10.1371/journal.pone.0282480 (PMC10019639; doi:10.1371/journal.pone.0282480)
Supplement: S1 Table — Note. Some Chinese MS words appeared more than once in the table or in different MS types because they are polysemous (Cheng et al., 2020; Tardif & Wellman, 2000). (DOCX) [file pone.0282480.s001.docx]

**Supporting Information**

**S1 Table. Examples of MS Talk Used by Western and Chinese Mothers.**

|  | English | Chinese |
| --- | --- | --- |
| **MS Type** |  |  |
| Cognition | “think”, “know”, “remember”, “reckon”, “wonder” | “觉得”, “知道”, “记得”, “认为”, “想” |
| Desire | “want”, “like”, “love”, “favorite” | “想/想要”, “喜欢”, “爱” |
| Emotion | “happy”, “upset”, “scared” | “高兴”, “开心”, “害怕”, “难过” |
| Modulations of assertions | “might”, “maybe”, “probably” | “可能”, “应该” |
| **Valence** |  |  |
| Positive | “enjoy”, “pleased”, “excited” | “高兴”, “开心”, “激动” |
| Negative | “sad”, “angry”, “frustrated” | “伤心”, “生气”, “害怕” |
| Neutral | “nervous” | “紧张” |

*Note.* Some Chinese MS words appeared more than once in the table or in different MS types because they are polysemous (Cheng et al., 2020; Tardif & Wellman, 2000).
